# Butterbur (*Petasites hybridus*) Extract Ameliorates Hepatic Damage Induced by Ovalbumin in Mice

**DOI:** 10.1155/2020/3178214

**Published:** 2020-12-15

**Authors:** Rana M. Alhusayan, Badr Abdullah Aldahmash, Doaa M. El-Nagar, Ahmad Rady, Khalid Elfakki Ibrahim, Saad Alkahtani

**Affiliations:** ^1^Department of Zoology, College of Science, King Saud University, P.O. Box 2455, Riyadh 11451, Saudi Arabia; ^2^Department of Zoology, Faculty of Women for Science, Arts and Education, Ain Shams University, Cairo, Egypt

## Abstract

The liver is the most vital organ that could be influenced by inducers of hypersensitivity such as ovalbumin. The current study was carried out to explore the effects of butterbur (*Petasites hybridus*) extract on the ovalbumin-induced liver hypersensitivity in Swiss albino male mice. Animals were divided into 4 groups, 1^st^ group served as a control group, 2^nd^ group treated with daily oral administration of 75 mg/kg of butterbur extract, 3^rd^ group received single oral dose 100 mg/kg of ovalbumin to induce hypersensitivity, and 4^th^ group treated with oral administration of butterbur extract one-day post to the hypersensitivity induction. Ovalbumin induces a significant increase in the activity of liver enzymes and MDA and decreased the activity of CAT after the ovalbumin treatment. Histopathological investigations revealed marked pathological alterations in liver tissues in the form of hyaline degeneration and fibrosis. Additionally, heavy immune response indicated by immunostaining of MDA and TNF-*α* could be observed. In contrast, posttreatment with butterbur extract after hypersensitivity induction resulted in a significant decrease of liver enzymes and oxidative stress and reduced the inflammation and fibrosis of liver tissues. These results suggest that butterbur extract is considered as anti-inflammatory and antioxidant therapeutic herb for hypersensitivity treatment of liver.

## 1. Introduction

The liver is the largest organ in the mammalian body that plays a main role in proteins, lipids, and carbohydrate metabolism [1]. Additionally, it plays a vital role to remove bacteria, viruses, and exogenous antigens from the systemic circulation. However, the liver might be subjected to injury associated with its role [2–5]. On the other hand, ovalbumin is known to induce asthma by causing chronic airway inflammation manifested by infiltration of neutrophils, eosinophils, and lymphocytes into bronchial lumen with increasing of reactive nitrogen and reactive oxygen species (ROS) levels. Inflammation and ROS production might induce liver injury, as well as lung damage ([6, 7]; Abdsmall et al., 2018).

Nowadays, herbal remedies attract growing interest as an alternative of chemical therapy, because of their potential role in reducing pathological changes and oxidative stress ([8–10];; [11]). *Petasites hybridus* or butterbur is considered one of the best remedies in treating asthma, cough, plague, and fever [12–14]. The main active constituents of butterbur are petasin and isopetasin [15]. Both are responsible for the inhibition of leukotriene synthesis and consequently, inflammation. Therefore, it had been suggested that *butterbur* is able to effectively treat urinary disorders, obstructive bronchitis, and bile obstruction, as well as liver and intestinal disorders [16, 17]. In this study, we illustrate the potential effect of *butterbur* extract to reduce the ovalbumin-induced inflammation and liver damages in albino mice.

## 2. Materials and Methods

### 2.1. Animals

Male mice of the Swiss albino strain aged 12 weeks and weighing 25 ± 5 g were used for the experiment. The animals were acclimated to 22(±1) °C and maintained under conditions of 12 h periods of light and dark, with free access to clean water and commercial mice food. The animals were housed in polypropylene cages inside a well-ventilated room. The experiments were approved by the board of studies, College of Science, King Saud University, Riyadh, Saudi Arabia. The animals which were used in this study were maintained and used by following the local and international guidelines for the use and care of laboratory animals.

### 2.2. Chemicals

Ovalbumin (cat # 02230) was purchased from LOBA CHEMIE, Colaba, Mumbai, Maharashtra 400005, India. Butterbur extract powder (*Petasites hybridus*) was from Swanson Health Products Company, 075 40th Ave S, Fargo, ND 58104, USA.

### 2.3. Experimental Design

Mice were distributed into four experimental groups each containing 10 mice. Animals were randomly divided into 4 groups, 1^st^ group served as the control group, 2^nd^ group treated with daily oral administration of 75 mg/kg of butterbur extract [18], 3^rd^ group received single oral dose 100 mg/kg of ovalbumin to induce hypersensitivity [19], and 4^th^ group treated with oral administration of butterbur extract one-day post to the hypersensitivity induction. Oral gavage was used for the administration of each of the medication. The experiment lasted for 45 days. At the end of the 45 days, the animals were sacrificed, and the blood was collected without anticoagulant and centrifuged at 3000 rpm for 15 minutes to separate the sera and stored at -80°C until used. Livers were collected, cut, and fixed in 10% neutral buffered formalin.

### 2.4. Liver Index

At the end of the experimental period, each mouse was weighed; livers were removed and weighed. Liver index was calculated by dividing the weight of liver by the body weight and then multiplying by 100 [20].

### 2.5. Biochemical Analysis

Sera were used for the estimation of ALT, AST, creatinine, and BUN according to Pratt and Kaplan [21].

### 2.6. Catalase (CAT)

Catalase activity in liver homogenate was assayed by the method according to [22]. The assay depends on the ability of the enzyme to inhibit phenazinemethosulphate-mediated reduction of nitro blue tetrazolium dye and measured at 560 nm (Bio-systems, BTS-350, Spain). The concentration of produced CAT has been expressed as nanomole per gram.

### 2.7. Lipid Peroxidation (MDA)

Lipid peroxidation in liver homogenate was determined according to the method of [23]. It determined by the absorbance at 535 nm (Bio-systems, BTS-350, Spain). The concentration of produced MDA has been expressed as nanomole per gram.

### 2.8. Histopathological Analysis

Livers were collected and cut into small pieces and fixed in 10% neutral buffered formalin. Following fixation, specimens were dehydrated, embedded in wax, and then sectioned into 5 *μ*m thickness sections. Staining was performed using hematoxylin/eosin and Masson's trichrome as described elsewhere [24]. Sections were examined under light microscope, and images were taken at 400x.

### 2.9. Immunohistochemical Analysis

The sections were prepared and mounted on electrostatic slides. Sections were deparaffinized in xylene and rehydrated in descending concentration of ethanol and finally in distilled water. Sections were heated in citrate buffer (pH = 6) within microwave for 5 minutes and washed with PBS buffer for 5 minutes. Sections were incubated in peroxidase blocking solution for 10 minutes and incubated overnight at 4°C in diluted primary antibodies (1 : 1000) anti-malondialdehyde (ab194225, Abcam) and anti-TNF-*α* antibody (ab1793, Abcam). Sections were rinsed with PBS buffer 3 times each for 5 minutes and incubated with biotinylated goat anti-mouse (ab128976) for 30 minutes at room temperature, followed by incubation in avidin-biotin complex for 30 min. The sections were then rinsed 3 times with PBS each for 5 minutes and incubated in DAB solution for 10 minutes. After that, Mayer's hematoxylin was added. Finally, the sections were dehydrated using ascending concentrations of ethanol and cleared with two washes of xylene. Sections were mounted, and images were taken at 400x. Images were analyzed using the Fiji software to calculate the optical density using the formula OD = log (max intensity/mean intensity), where the max intensity = 255 for 8-bit images.

### 2.10. Statistical Analysis

One-way ANOVA was carried out, and the statistical comparisons among the groups were performed with Duncan's test using a statistical package program (SPSS version 16.0). Data = mean ± standard error of mean and *p* values <0.05 were considered significant.

## 3. Results

### 3.1. Posttreatment with Butterbur Extract Decreases the Liver Index of Ovalbumin-Treated Mice

**Figure 1:**
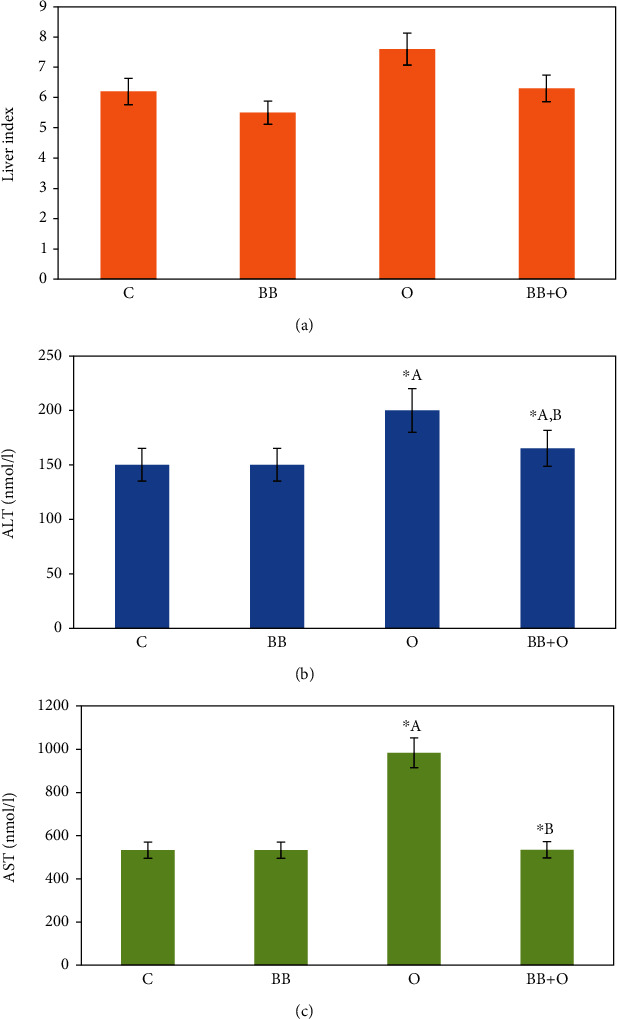
Effects on liver index and liver functions. (a) Histogram of liver index showing a significant increase of the group treated with 100 mg/kg ovalbumin compared to the control group and a significant decrease in the posttreated group with 75 mg/kg of butterbur extract. (b, c) Ovalbumin (100 mg/kg) induces a significant increase of ALT and AST activities of treated mice, respectively, compared to the control mice. Posttreatment with butterbur extract (75 mg/ml) induces a significant decrease of both activities. Data = mean ± the standard error of mean. ^∗^^a^Significant compared to the control group. ^∗^^b^Significant compared to the ovalbumin group. *p* ≤ 0.05.

To investigate the effect of butterbur extract on the ovalbumin-induced liver abnormalities in mice, liver index has been calculated. As shown in (Figure 1(a)), we could observe an insignificant decrease in liver index in butterbur-treated animals in comparison to the untreated controls. In contrast, ovalbumin-treated animal showed an increase in liver index in comparison to the control animals. However, posttreatment with butterbur (75 mg/kg) insignificantly decreases the liver index of the ovalbumin-treated animals.

### 3.2. Butterbur Extract Improves Liver Function of Ovalbumin-Treated Mice

Liver function in mice was assessed by measuring the level of liver enzymes (ALT and AST) in treated mice and control group. The group that received butterbur extract showed insignificant changes compared to the control group in all tested animals. In contrast, ovalbumin administration induced a significant increase of ALT and AST activities (Figures 1(b) and 1(c), respectively), whereas, posttreatment with butterbur seems to rescue the liver toxicity induced by ovalbumin. Indeed, ALT and AST activities dropped significantly compared to ovalbumin-treated mice as shown in Figures 1(b) and 1(c), respectively.

### 3.3. Butterbur Extract Lowers the Ovalbumin-Induced Oxidative Stress in Mice Liver

**Figure 2:**
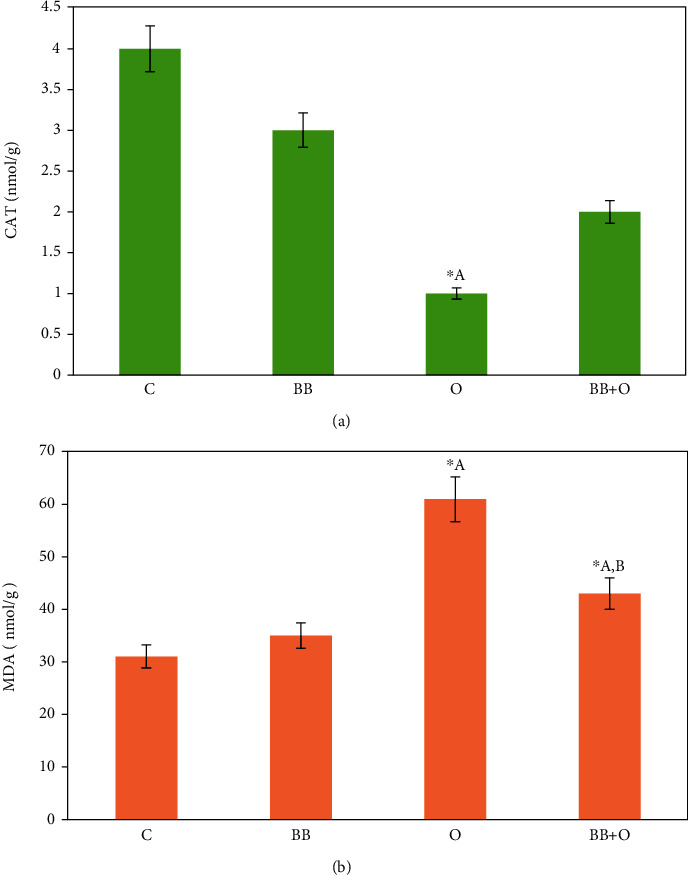
Butterbur extract lowers oxidative stress induced by the ovalbumin in mice liver. Histograms of oxidative stress activity. (a) Ovalbumin treatment (100 mg/kg) shows a significant decrease of CAT activity compared to control and an insignificant increase in the posttreated mice group with 75 mg/kg of butterbur extract. (b) Ovalbumin treatment (100 mg/kg) reveals a significant increase of MDA activity compared to the control group. Posttreatment with butterbur extract (75 mg/kg) displays a significant decrease of MDA activity. Data = mean ± the standard error of mean. ^∗^^a^Significant compared to the control group. ^∗^^b^Significant compared to the ovalbumin group. *p* ≤ 0.05.

We further investigated the effect of butterbur extract on the ovalbumin-treated mice. The oxidative stress level was detected by measuring the known oxidative stress markers CAT and MDA in liver homogenate. The results are shown in Figure 2. Mice livers treated with butterbur extract displayed an insignificant change in CAT and MDA activities compared to the untreated control group. In contrast, we measured a significant decrease of CAT activity, as well as a significant increase of MDA activity in ovalbumin-treated mice compared to the untreated control group, whereas posttreatment of butterbur extract resulted in an insignificant increase of CAT activity and a significant decrease of MDA activity compared to ovalbumin-treated group that referred to the effective role of butterbur extract to lower the oxidative stress induced by ovalbumin.

### 3.4. Butterbur Extract Posttreatment Improves the Pathological Abnormalities Induced by Ovalbumin Treatment in Mice Liver

**Figure 3:**
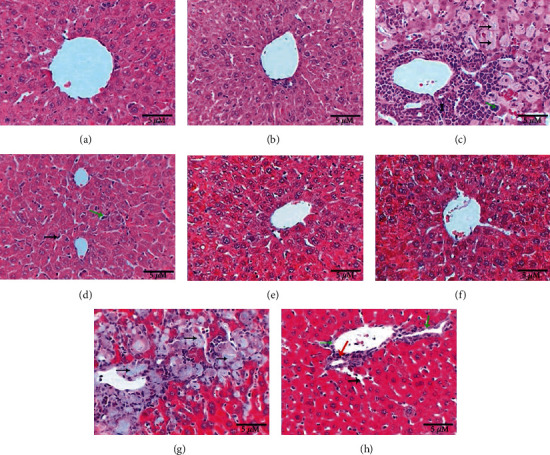
Posttreatment with butterbur extract reduces the pathological singe induces after ovalbumin treatment. Photomicrographs of liver showing (a) normal control liver; (b) normal structure of liver in the group treated with butterbur extract (75 mg/kg); (c) liver of the group treated with (100 mg/kg) ovalbumin shows great aggregations of inflammatory cells (labelled I), giant macrophage (green arrow), and hyaline degeneration (black arrows); (d) liver of the group posttreated with butterbur extract shows healthy hepatocytes, activation of Kuppfer cells (black arrow), and inflammatory cells (green arrows) (H&E-400x scale 10 *μ*m); (e) no fibrosis in control liver; (f) no collagenous fibers in the liver of the group treated with butterbur; (g) liver of the group treated with ovalbumin (100 mg/kg) reveals depositions of collagenous fibers surrounded the vein and in between hyaline-degenerated hepatocytes (black arrows); (h) liver of the group posttreated with butterbur extract (75 mg/kg) displays less depositions of collagenous fibers (green arrows), inflammatory cells (red arrows), and activation of Kuppfer cells (black arrows) (M.Tr.-400x; scale bar = 10 *μ*m).

We further explored the effect of butterbur extract on the ovalbumin-induced toxicity using histochemical analysis of treated and nontreated liver sections. As shown in (Figure 3(a)), control liver section exhibited normal liver tissue that consisted of central vein surrounded by network of hepatocytes separated from each other by blood sinusoids with Kupffer cells. Similar results could be obtained in butterbur-treated animals. However, activation of Kupffer cells could be observed here (Figure 3(b)). In contrast, liver section of mice treated with ovalbumin exhibited marked pathological abnormalities, manifested by accumulation of large infiltration cells and giant macrophages. Furthermore, hyaline degeneration, fibrosis, and necrotic foci could be also observed (Figure 3(c)). In contrast, posttreatment with butterbur extract revealed healthy hepatocytes with some Kupffer cell activation and a few numbers of scattered inflammatory cells (Figure 3(d)).

On the other side, Masson's trichrome-stained liver sections revealed no collagenous fiber depositions in both control and mice group treated with butterbur extract (Figures 3(e) and 3(f)), whereas the liver section treated with ovalbumin displayed heavy incidence of collagenous fiber depositions between hyaline-degenerated hepatocytes and around the vein (Figure 3(g)). Posttreatment of butterbur extract showed less depositions of collagenous fibers surrounding the vein and a few numbers of inflammatory cells (Figure 3(h)).

### 3.5. Butterbur Extract Posttreatment Lowers the Ovalbumin-Induced Oxidative Stress and Inflammation in Mice Liver

**Figure 4:**
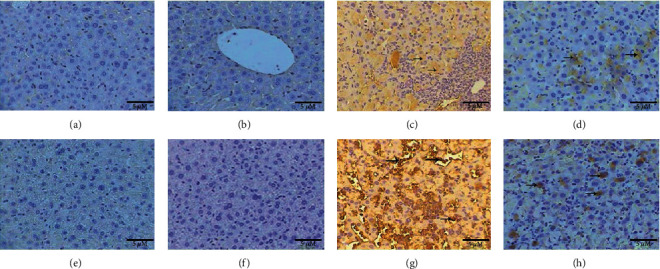
Posttreatment with butterbur extract reduces MDA and TNF-*α* in ovalbumin-treated liver. Photomicrographs of liver sections stained immunohistochemically (a) no immune response against MDA in the control group, (b) no immune response in the group treated with butterbur extract (75 mg/kg), (c) intense immune response against MDA (arrows) in ovalbumin-treated group, (d) weak immune response against MDA in the group posttreated with butterbur extract to ovalbumin, (e) no immune response against TNF-*α* in the control group, (f) no immune response in the group treated with butterbur extract, (g) strong immune reactivity against TNF-*α* in the group treated with ovalbumin, and (h) weak immune reactivity against TNF-*α* in the group posttreated with butterbur extract to ovalbumin (ABC-method 400x; scale bar = 10 *μ*m).

**Figure 5:**
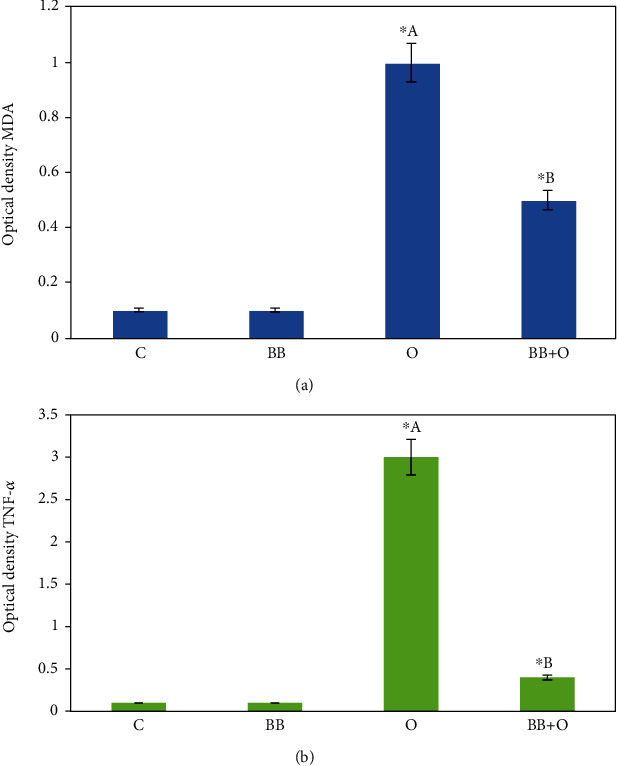
Histograms of optical density of immunohistochemistry staining. (a) Optical density of images stained against MDA shows a significant increase in the group treated with 100 mg/kg of ovalbumin and a significant decrease in the posttreated group with 75 mg/kg of butterbur extract. (b) Optical density of images stained against TNF-*α* shows a significant increase in the group treated with 100 mg/kg of ovalbumin and a significant decrease in the posttreated group with 75 mg/kg of butterbur extract. Data = mean ± the standard error of mean. ^∗^^a^Significant compared to the control group. ^∗^^b^Significant compared to the ovalbumin group. *p* ≤ 0.05.

As shown in Figures 4(a) and 4(b), control group and butterbur-treated groups showed no immune response against MDA and TNF-*α* antibodies, whereas the group treated with ovalbumin revealed strong immune response manifested by intense brown spots against MDA (Figure 4(c)) and also against TNF-*α*. Interestingly, the last spots were mainly concentrated in leukocytes (Figure 4(g)). In addition to a significant increase of optical density compared to the control group (Figure 5), butterbur extract posttreatment resulted in a weak immune response against MDA (Figure 4(d)) and TNF-*α* (Figure 4(h)). Furthermore, optical density revealed a significant decrease compared to the ovalbumin-treated group (Figure 5).

Collectively, our results suggest a positive effect of butterbur extract on the reduction of oxidative stress and inflammation induced by ovalbumin.

## 4. Discussion

Ovalbumin has been reported to develop hypersensitivity in lung tissues indicated by perbronchial and perivascular cell inflammation composed principally of eosinophils and lymphocytes [25]. Additionally, ovalbumin induces hypersecretion of mucus in treated animals, which considers the prominent histopathological features of ovalbumin-induced hypersensitivity [26]. Furthermore, it considered as a potent allergen that causes activation of allergic mediators such as mast cells, inflammatory cytokines, eosinophils, and histamine, which have been recognized to play a key role in inflammatory reactions against ovalbumin upon exposure [27, 28]. To our knowledge, the induction of hypersensitivity through ovalbumin in the liver has not been reported. Therefore, the biochemical and pathological changes in the liver after ovalbumin treatment had been highlighted for the first time in this study. The ovalbumin treatment exhibited high levels of liver enzymes due to severe degeneration of hepatocytes, besides accumulation of large aggregations of inflammatory cells and macrophages, in addition to the induction of hyaline degeneration, necrosis, and fibrosis.

Some studies have indicated that allergens in general lead to autoimmune inflammation in the liver such as granulocytic and lymphocytic cell inflammation, granulomas, necrosis, and fibrosis [6, 29, 30]. Indeed, the present study illustrated that ovalbumin induced hypersensitivity in the form of heavy oxidative stress in treated mice. This hypersensitivity is manifested by the high level of MDA activity and the significant decrease of CAT activity in liver homogenate. Additionally, the immunohistochemistry study showed high incidence of MDA in the liver tissue and strong immune reactivity against TNF-*α*.

Butterbur is a herbaceous plant, belonging to the *Asteraceae*, that has been used in folk medicine in Asia and America for therapeutic of many illnesses such as fever, respiratory diseases, spasms, and pain. Beside many other compounds included in butterbur, two major compounds (petasin and isopetasin) are suggested to be responsible for the pharmacological effects of butterbur [31, 32]. The extracts from butterbur are used to treat a variety of ailments including spasms of the urogenital and digestive tracts, asthma, migraines, and allergic rhinitis [33–35]. The current study pointed out that butterbur extract is a potent inhibitor of hypersensitivity induced by ovalbumin. This finding had been suggested depending on the decrease of hypersensitivity-related liver enzymes in response to butterbur extract treatment. Catalase is an important liver enzyme that is dealing with oxidative stress and reactive oxygen species. Ovalbumin reduced the activity of CAT in treated mice; however, posttreatment with butterbur extract reinduces the CAT activity. The activity CAT in addition to decreasing of MDA and increasing of CAT activities that changed due to ovalbumin administration that coincided with findings of other study demonstrated that butterbur extract could enhance the biochemical and oxidative stress which changes by kainic acid [36]. It was reported that the intense antioxidant effect of butterbur extract might be attributed to the impact incidence of polyphenols, glycosides, and some flavonoids as patuletin. On the other hand, the presence of furofuran lignan called petaslignolide seemed to have powerful antioxidant effect that exerts an effort to ameliorate the hepatocellular damage by reducing oxidative stress [37]. Moreover, treatment with butterbur extract after hypersensitivity induction resulted in attenuation of pathological signs resulted from hypersensitivity as inflammation, degeneration, and fibrosis that agreed with previous studies which clarified that petasin and isoptasin of butterbur could exert anti-inflammatory actions via inhibition of leukotriene synthesis and cyclooxygenases [12]. Furthermore, decreasing of oxidative stress and inflammation in the liver tissue was clarified by immunohistochemistry. Altogether, our study suggests the effectiveness of butterbur extract in the treatment of ovalbumin-induced hypersensitivity in mice liver.

## 5. Conclusion

Present finding suggests that butterbur extract is considered as an anti-inflammatory and an antioxidant therapeutic herb for hypersensitivity treatment of liver.

## Data Availability

The data generated or analyzed in this article are online publicly available without request.
